# Characterisation of the *ERF102* to *ERF105* genes of *Arabidopsis thaliana* and their role in the response to cold stress

**DOI:** 10.1007/s11103-020-00993-1

**Published:** 2020-03-18

**Authors:** Sylvia Illgen, Stefanie Zintl, Ellen Zuther, Dirk K. Hincha, Thomas Schmülling

**Affiliations:** 1 Institute of Biology/Applied Genetics, Dahlem Centre of Plant Sciences (DCPS), Freie Universität Berlin, Albrecht-Thaer-Weg 6, 14195 Berlin, Germany; 2 Max-Planck-Institute of Molecular Plant Physiology, 14476 Potsdam, Germany

**Keywords:** *Arabidopsis thaliana*, Cold acclimation, *ETHYLENE RESPONSE FACTOR* genes, Freezing tolerance, Root architecture, Transcription factor

## Abstract

**Key message:**

The four phylogenetically closely related ERF102 to ERF105 transcription factors of *Arabidopsis thaliana* are regulated by different stresses and are involved in the response to cold stress.

**Abstract:**

The *ETHYLENE RESPONSE FACTOR* (*ERF*) genes of *Arabidopsis thaliana* form a large family encoding plant-specific transcription factors. Here, we characterise the four phylogenetically closely related *ERF102/ERF5*, *ERF103/ERF6*, *ERF104* and *ERF105* genes. Expression analyses revealed that these four genes are similarly regulated by different hormones and abiotic stresses. Analyses of tissue-specific expression using *promoter:GUS* reporter lines revealed their predominant expression in root tissues including the root meristem (*ERF103*), the quiescent center (*ERF104*) and the root vasculature (all). All GFP-ERF fusion proteins were nuclear-localised. The analysis of insertional mutants, amiRNA lines and *35S:ERF* overexpressing transgenic lines indicated that *ERF102* to *ERF105* have only a limited impact on regulating shoot and root growth. Previous work had shown a role for ERF105 in the cold stress response. Here, measurement of electrolyte leakage to determine leaf freezing tolerance and expression analyses of cold-responsive genes revealed that the combined activity of ERF102 and ERF103 is also required for a full cold acclimation response likely involving the CBF regulon. These results suggest a common function of these *ERF* genes in the response to cold stress.

**Electronic supplementary material:**

The online version of this article (10.1007/s11103-020-00993-1) contains supplementary material, which is available to authorized users.

## Introduction

The *ERF* genes encode plant-specific transcription factors forming a large gene family with 122 members in *Arabidopsis thaliana* (Nakano et al. 2006). The ERF transcription factors are members of the APETALA2/ETHYLENE RESPONSE FACTOR (AP2/ERF) superfamily, which also contains the AP2 and RAV families and which is defined by the AP2/ERF DNA-binding domain (Riechmann et al. 2000). This domain is about 60 amino acids long and forms an interface of three antiparallel β-strands and one α-helix (Ohme-Takagi and Shinshi 1995). The β-strands bind to an 11 bp consensus sequence (5′-TAAGAGCCGCC-3′), the GCC-Box, in the major groove of the DNA double helix (Hao et al. 1998). ERF transcription factors are involved in the regulation of numerous developmental processes (Riechmann and Meyerowitz 1998) and they are important for the response to various biotic and abiotic stresses including cold (Kizis et al. 2001; Agarwal et al. 2006b; Srivastava and Kumar 2019; Xie et al. 2019).

Previously, we identified four phylogenetically closely related *ERF* genes with similar transcriptional responses to cytokinin (Brenner et al. 2005). These genes, *ERF102* (AT5G47230; known as *ERF5*), *ERF103* (AT4G17490; identical to *ERF6*), *ERF104* (AT5G61600) and *ERF105* (AT5G51190) are members of group IXb of the ERF family (Nakano et al. 2006). Expression of *ERF102* to *ERF105* is regulated by cold and different cold stress-related hormones, and it was demonstrated that *ERF105* has a function in the freezing tolerance and cold acclimation of *Arabidopsis* (Bolt et al. 2017). All four *ERF* genes are also involved in the response to other stresses*. ERF102* and *ERF103* regulate leaf growth inhibition upon mild osmotic stress (Dubois et al. 2013, 2015) and *ERF103* additionally regulates oxidative stress responses (Sewelam et al. 2013). *ERF103*, *ERF104* and *ERF105* are involved in the fast retrograde signalling response and the acclimation response to high light (Moore et al. 2014a, b; Vogel et al. 2014). Further studies have shown that *ERF102* to *ERF105* play a role in plant immunity (Bethke et al. 2009; Moffat et al. 2012; Son et al. 2012; Mase et al. 2013; Meng et al. 2013; Cao et al. 2019). Thus, ERF102 to ERF105 match the profile of other ERF transcription factors designated as a regulatory hub integrating hormone signalling in the plant response to abiotic stresses (Müller and Munné-Bosch 2015).

The close phylogenetic relationship among the four *ERF* genes and the similarity of their transcriptional responses to different cues suggested that they share some common functions in response to cold. Cold stress adversely affects plant growth and development and several pathways to respond to cold stress have been described. Plants from temperate and boreal climates have evolved mechanisms to acquire freezing tolerance through cold acclimation, a process in which upon exposure to low non-freezing temperatures the ability to survive freezing temperatures increases (Xin and Browse 2000). A central cold signalling pathway is the CBF (*C-REPEAT-BINDING FACTOR/DEHYDRATION-RESPONSE ELEMENT-BINDING PROTEIN*) regulon. The *CBF1* (*DREB1b*), *CBF2* (*DREB1c*) and *CBF3* (*DREB1a*) genes are the central regulatory elements of this regulon (Liu et al. 1998; Chinnusamy et al. 2007). The INDUCER OF C-REPEAT-BINDING FACTOR EXPRESSION 1 (ICE1), a MYC-type bHLH (basic helix-loop-helix) transcription factor, is post-translationally activated in response to cold (Chinnusamy et al. 2003; Miura et al. 2007; Ding et al. 2015; Li et al. 2017). ICE1 in turn activates the transcription of the *CBF3* gene (Chinnusamy et al. 2003). Besides ICE1, expression of the cold-regulated *CBF* genes is positively controlled by several other transcription factors including ICE2 and CALMODULIN-BINDING TRANSCRIPTION ACTIVATOR 3 (CAMTA3) (Doherty et al. 2009; Fursova et al. 2009). Negative regulators of the CBF regulon are, for instance, the C2H2 zinc finger transcription factor ZAT12 (Vogel et al. 2005) and MYB15 (Agarwal et al. 2006a). MYB15 is in turn negatively regulated by ICE1 (Agarwal et al. 2006a) and phosphorylation of MYB15 by MPK6 reduces its affinity to bind to the *CBF3* promoter (Kim et al. 2017). The CBF proteins regulate the expression of the *COLD-REGULATED* (*COR*) genes and physiological responses (e.g. accumulation of cryoprotective compounds, modification of cellular structures) that together confer cold acclimation (Thomashow 1999; Yamaguchi-Shinozaki and Shinozaki 2006). Transcriptomic analyses of the CBF regulon has revealed that only part (~ 11%) of the cold-responsive genes is under control of the CBF regulon (Park et al. 2015), which was confirmed by gene expression analysis in *cfb* triple mutants (Jia et al. 2016; Zhao et al. 2016). It was concluded that only about one-third of the increase in freezing tolerance that occurs in response to low temperature is dependent on the CBF regulon (Park et al. 2015). Together, this suggests that an extensive regulatory network involving numerous transcription factors in addition to the best known CBF core regulators governs the response to cold.

We previously identified the *ERF105* gene of *Arabidopsis* as an important factor for *Arabidopsis* freezing tolerance and cold acclimation (Bolt et al. 2017). The strongly reduced expression of cold-responsive genes in *ERF105* mutants upon cold acclimation suggests that its action is linked to the CBF regulon. Also the expression of three closely related transcription factor genes, *ERF102*, *ERF103* and *ERF104*, is induced by cold (Lee et al. 2005; Vogel et al. 2005; Park et al. 2015; Bolt et al. 2017). It is therefore possible that these transcription factors have a function in the response to cold stress. Here, we have extended our analysis of the *ERF105* gene family. We provide additional transcript data supporting a similar response profile of the *ERF105* family members and show the tissue-specific expressions of *pERF102:GUS* to *pERF104:GUS* as well as the subcellular localisations of GFP-ERF102 to GFP-ERF104 fusion proteins. Single and combined loss-of-function mutants and lines overexpressing single *ERF* genes were analysed for their growth characteristics and cold stress response and reveal partially similar functions of the members of this transcription factor subfamily.

## Results

### Phylogenetic analysis and description of the ERF102 to ERF105 proteins of *Arabidopsis thaliana*

**Fig. 1:**
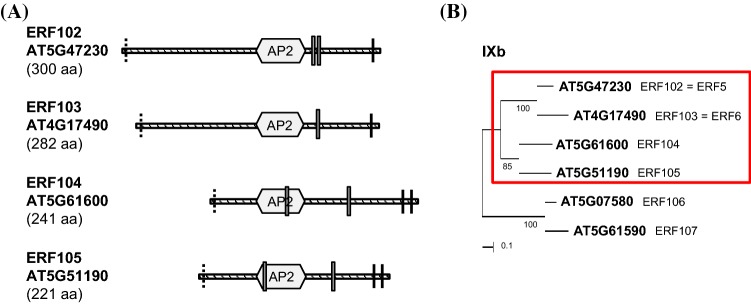
Description of the ERF102 to ERF105 proteins of *Arabidopsis thaliana*. (**a**) Structure of the *Arabidopsis* ERF102 to ERF105 proteins. The schematic representation shows the protein structures of ERF102 to ERF105 according to Nakano et al. (2006). The striped lines represent the protein sequences, the hexagons indicate the AP2/ERF DNA-binding domain, black lines putative phosphorylation sites, dashed lines the putative transactivation domains (Nakano et al. 2006) and grey boxes the nuclear localisation signals determined with WoLF PSORT (Horton et al. 2007). (**b**) An unrooted phylogenetic tree of group IXb ERF transcription factors showing the close evolutionary relationship between ERF102 to ERF105 (red box) that are studied. The phylogenetic tree was constructed using MEGA6, the numbers indicate bootstrap values (Tamura et al. 2013)

According to 'The *Arabidopsis* Information Resource' (TAIR) (Huala et al. 2001), *ERF102* to *ERF105* are relatively small, intronless genes with coding regions for proteins containing 300 (ERF102), 282 (ERF103), 241 (ERF104) and 221 (ERF105) amino acids. Like all AP2/ERF transcription factors they possess the characteristic AP2/ERF domain and are the only proteins in group IX with one (ERF102 and ERF103) or two (ERF104 and ERF105) putative phosphorylation sites (Nakano et al. 2006). Moreover, ERF102 to ERF105 possess acidic regions that might function as transcriptional activation domains (Fujimoto et al. 2000). According to WoLF PSORT (Horton et al. 2007) ERF103 has a single nuclear localisation signal (NLS) whereas ERF102, ERF104 and ERF105 have two NLS (Fig. 1a).

Comparison of the amino acid sequences of ERF102 to ERF105 using MUSCLE (Edgar 2004) revealed a sequence identity of 40% between all four proteins with high conservation of the AP2/ERF domain. The protein pairs share 67% (ERF102 and ERF103) and 52% (ERF104 and ERF105) amino acid identity. Phylogenetic analysis confirmed that ERF102 to ERF105 are closely related, with ERF102 and ERF103 together on one branch and ERF104 and ERF105 on the other branch of the phylogenetic tree (Fig. 1b).

### The *ERF102* to *ERF105* transcription factor genes show a similar transcriptional regulation pattern

**Fig. 2:**
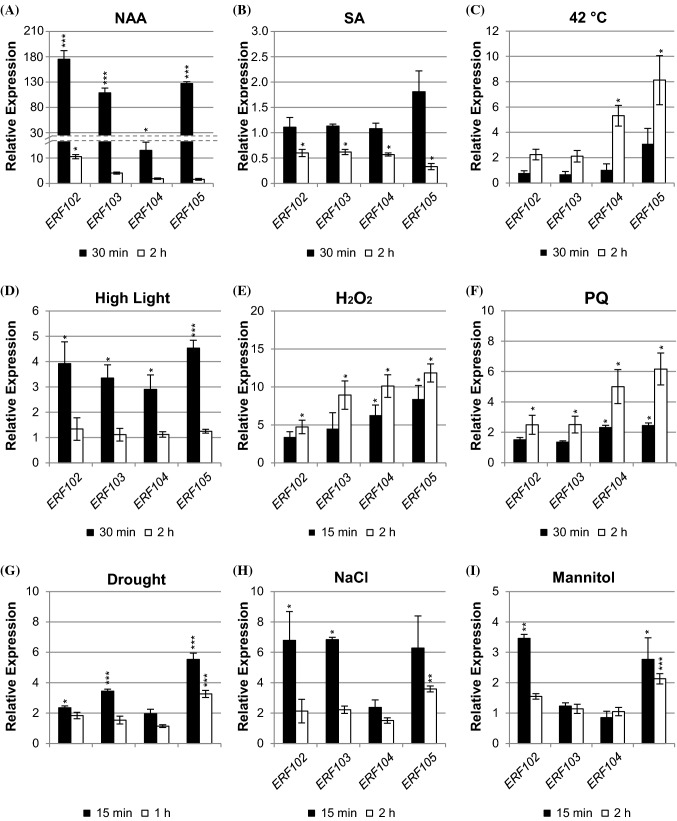
Regulation of *ERF102* to *ERF105* gene expression. Relative expression of *ERF102* to *ERF105* in eleven-day-old wild-type seedlings (eight pooled seedlings per sample) after hormone or stress treatment. **a** Auxin (10 µM NAA), **b** salicylic acid (10 mM SA), **c** heat (42 °C), **d** high light (1000 µmol m^−2^ s^−1^), **e** and **f** oxidative stress (**e**; 500 mM H2O2, **f**; 30 µM paraquat), **g** drought, **h** salt (200 mM NaCl) and **i** osmotic stress (200 mM mannitol). Transcript levels of wild-type samples under control conditions were set to 1 (n ≥ 4). Asterisks indicate significant differences to the respective mock treatment (*p < 0.05; **p < 0.01; ***p < 0.001). Error bars represent SE

Analysis of transcriptional regulation may yield indications on functional context, therefore the previous work showing that *ERF102* to *ERF105* are regulated similarly by cold and different cold stress-related hormones, including ethylene, jasmonate and abscisic acid (Bolt et al. 2017), was extended. First we complemented the comparison of the hormonal transcriptional regulation of the four *ERF* genes and analysed their response to auxin and salicylic acid (SA). Auxin (NAA) rapidly and strongly induced the transcript abundances of all four *ERF* genes about 180-fold (*ERF102*), 100-fold (*ERF103*), 13-fold (*ERF104*) and 130-fold (*ERF105*) after 30 min. This increase was transient as 2 h after auxin treatment the transcript abundances were only increased between 11-fold (*ERF102*) and twofold (*ERF105*) (Fig. 2a). In contrast, the transcript levels of all four *ERF* genes were downregulated by SA to about 50% of the initial level after 2 h (Fig. 2b).

Next, the response to different stress treatments was studied. Heat stress (42 °C) induced an upregulation of *ERF104* and *ERF105* of about fivefold and eightfold, respectively, after 2 h (Fig. 2c). High light (1000 µmol m^−2^ s^−1^) provoked a rapid upregulation of all four genes about fourfold (*ERF102*), threefold (*ERF103* and *ERF104*) and 4.5-fold (*ERF105*) after 30 min. The transcripts were back to their initial levels after 2 h (Fig. 2d). Oxidative stress imposed by H_2_O_2_ treatment resulted in a rapid upregulation of all four genes after 15 min by about 3.5-fold (*ERF102*), 4.5-fold (*ERF103*), 6.5-fold (*ERF104*), and 8.5-fold (*ERF105*). After 2 h transcript levels were increased further to about fivefold (*ERF102*), ninefold (*ERF103*), tenfold (*ERF104*) and 12-fold (*ERF105*) compared to the initial level (Fig. 2e). Oxidative stress imposed by treatment with the superoxide-generating herbicide paraquat showed a similar result (Fig. 2f). A fast transcriptional response of the *ERF* genes was also observed after drought stress that led to an about twofold (*ERF102* and *ERF104*), 3.5-fold (*ERF103*) and 5.5-fold (*ERF105*) upregulation of transcript levels within 15 min, which were decreased again after 1 h (Fig. 2g). Salt stress (200 mM NaCl) also caused a rapid but transient upregulation of the *ERF* genes up to about six- to seven-fold for the *ERF102*, *ERF103* and *ERF105* genes (Fig. 2h). Two of the genes (*ERF102*, *ERF105*) also responded rapidly to mannitol application (Fig. 2i).

We have analyzed the occurrence and distribution of *cis*-acting response elements (CAREs) in 2 kb of the promoters 5′ upstream of all four *ERF* genes. Table S1 shows the frequency of a representative but non-exhaustive list of CAREs functionally related to the regulation of the *ERF102* to *ERF105* genes by hormonal and environmental cues documented in Fig. 2 and Bolt et al. (2017). Fig. S1 illustrates the distribution of a selection of these CAREs along the promoters of the four *ERF* genes revealing distinct patterns despite the often similar transcriptional responses to hormones and stress treatments.

Taken together, the four *ERF* genes showed similar, very rapid and often transient transcriptional responses to different plant hormones, including an extraordinarily strong induction by auxin, as well as rapid, strong and often comparable responses to different stress treatments. Some individual response profiles such as stronger responses to heat by *ERF104* and *ERF105* or the lack of response to NaCl and mannitol by *ERF104* were observed as well. These partly similar stress response profiles would be consistent with overlapping functions in response to these stresses.

### *pERF102:GUS* to *pERF105:GUS* reporter genes are expressed in different tissues in *Arabidopsis thaliana*

Transgenic plants expressing the *GUS* reporter gene under the control of ~ 2 kb of the *ERF102* to *ERF104* promoters located 5´ upstream of the coding regions were analysed to determine the tissue-specific expression of these genes.

**Fig. 3:**
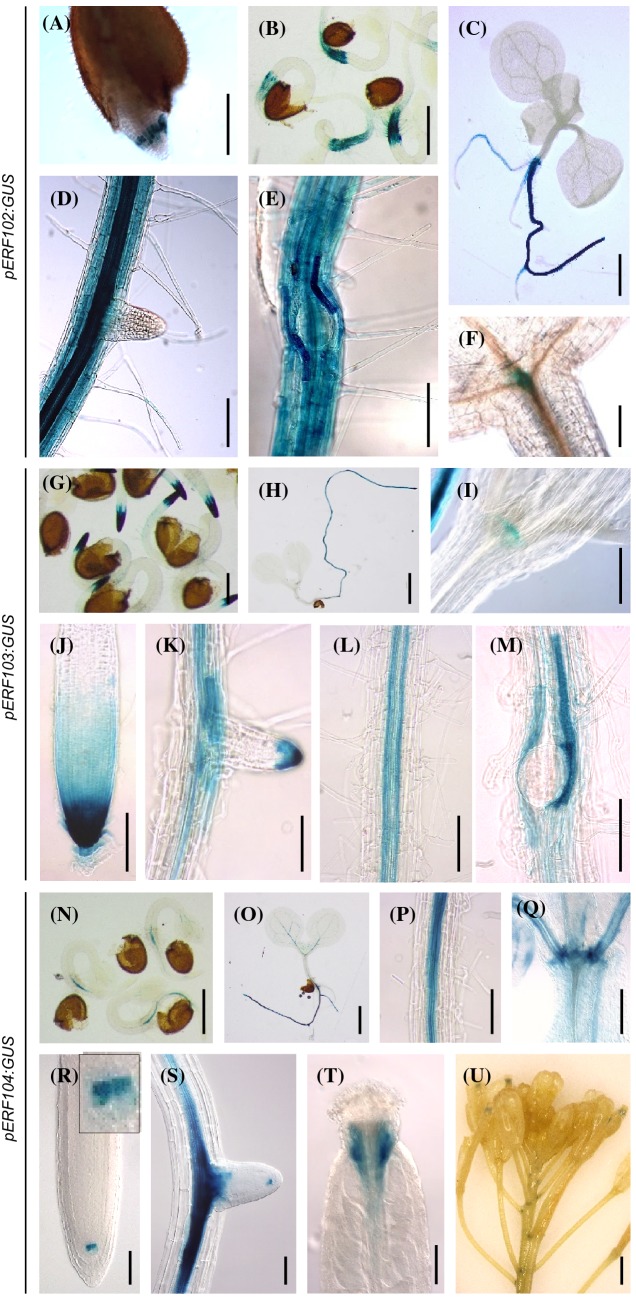
Expression of the *GUS* reporter gene under control of the *ERF102*, *ERF103* and *ERF104* promoters. Histochemical localisation of GUS activity in *Arabidopsis pERF:GUS* reporter lines. *pERF102:GUS* seedlings 30 h (**a**) and 60 h (**b**) after imbibition of seeds and ten DAG (**c‒f**). **a**, **b** Germinating seeds, **c** whole seedling, **d** and **e** primary root with emerging lateral roots and **f** shoot apex with a stained apical meristem. *pERF103:GUS* seedlings 60 h (**g**) after imbibition of seeds and seven DAG (**h‒m**). **g** Germinating seeds, **h** whole seedling, **i** shoot apex with stained shoot apical meristem, **j** tip of primary root, **k** lateral root, **l** vasculature of primary root, **m** primary root with emerging lateral root. *pERF104:GUS* seedlings 60 h (**n**) after imbibition of seeds, seven (**o–s**) and 21 DAG (**t**, **u**). **n** Germinating seeds, **o** whole seedling, **p** vasculature of primary root, **q** shoot apex, (inset shows stained quiescent center cells), **r** tip of primary root, **s** primary root with emerging lateral root, **t** apical part of gynoecium with stained style and **u** inflorescence with flowers and young siliques. Scale bars = 100 µm in **a**, **d**, **e**, **i‒m**, **p**, **q**, and **u**; 400 µm in **b** and **n**; 1 mm in **c**; 50 µm in **f**, **r** and **s**; 200 µm in **g**; 2 mm in **h**, **o** and **t**

Thirty h after imbibition, strong GUS activity of *pERF102:GUS* plants was detected in the root tip transition zone of germinated seedlings (Fig. 3a) and expanded within the next 30 h within the radicle (Fig. 3b). Ten DAG, *pERF102:GUS* was expressed in all root tissues except root tips and root hairs. The strongest GUS activity was observed in the vascular bundle of primary roots and in cortex cells that surround emerging lateral roots (Fig. 3c‒e). Weak *pERF102:GUS* expression was detected in the shoot apical meristem (SAM) of seedlings (Fig. 3f).

*pERF103:GUS* activity was detected 60 h after imbibition in the root tip (Fig. 3g) and seven DAG in the whole root (Fig. 3h). Very high activity was detected in the root apical meristem (RAM) (Fig. 3j). *pERF103:GUS* was also expressed in the root tip of lateral roots, but only after stage VIII of lateral root development (Péret et al. 2009) (Fig. 3k). GUS activity was observed in the vasculature of primary roots (Fig. 3l), but not in the vasculature of emerging or fully developed lateral roots, and in cortex cells that surround emerging lateral roots (Fig. 3m). In shoot tissues, weak expression of *pERF103:GUS* was detected only in the shoot apex (Fig. 3i).

*pERF104:GUS* expression was also detected early after germination. Sixty h after imbibition, *pERF104:GUS* was weakly expressed in the vasculature of hypocotyls and cotyledons and slightly stronger in the vasculature of radicles (Fig. 3n). Seven-day-old seedlings showed GUS activity in the vascular tissues as well as in the shoot apex (Fig. 3o‒q). A particularly well-defined local GUS signal was noted in the quiescent center of roots (Fig. 3r, s). In addition, GUS activity was detected in the style of the gynoecium and at the base and in the apex of siliques (Fig. 3t, u).

As plants matured, GUS activity of *pERF102:GUS* to *pERF104:GUS* plants was present in the same tissues as in young seedlings but declined progressively (data not shown). Together, *promoter:GUS* fusions of all three *ERF* genes were predominantly expressed in root tissues, similar to *pERF105:GUS* (Bolt et al. 2017).

### GFP-ERF102 to GFP-ERF105 are located in the nucleus

**Fig. 4:**
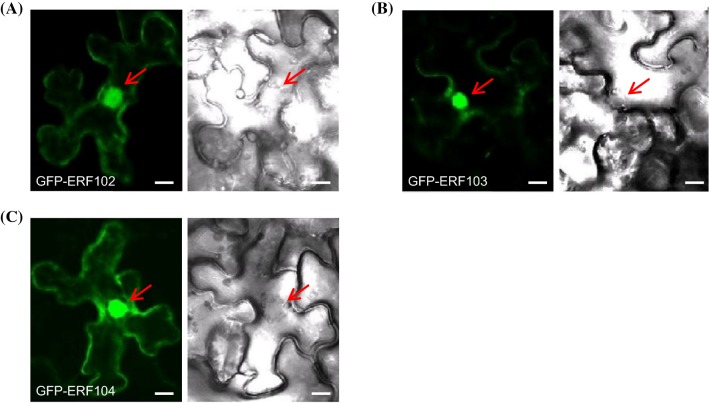
Subcellular localisation of GFP-ERF102, GFP-ERF103 and GFP-ERF104 fusion proteins. Transient expression of (**a**) *35S:GFP-ERF102,* (**b**) *35S:GFP-ERF103* and (**c**) *35S:GFP-ERF104* in leaf epidermis cells of *N. benthamiana* was analysed by confocal laser scanning microscopy. Left, fluorescence of GFP; right, bright field picture. The red arrows indicate the nucleus. Scale bars = 10 µm

To examine the subcellular localisation of the ERF102 to ERF104 proteins, full-length cDNAs of *ERF102* to *ERF104* were fused in frame to the 3′ end of the *GREEN FLUORESCENT PROTEIN* (*GFP*) coding sequence. The resulting *GFP-ERF102*, *GFP-ERF103* and *GFP-ERF104* fusion genes driven by the cauliflower mosaic virus (CaMV) *35S* promoter were transiently expressed in *Nicotiana benthamiana* leaf cells. Confocal imaging of GFP fluorescence in leaf cells showed that all three fusion proteins were predominantly located in the nucleus, weaker signals were derived from the cytosol (Fig. 4). This pattern was similar to the predominant nuclear localisation of GFP-ERF105 (Bolt et al. 2017).

### Characterisation of plants with altered *ERF102* to *ERF105* expression levels

To identify and compare biological functions of the *ERF102* to *ERF104* genes, we studied transgenic lines with altered expression levels. For *ERF102*, a homozygous T-DNA insertion line (*erf102*; SAIL_46_C02) was obtained. Verification of the annotated location of the T-DNA insertion in *erf102* by sequencing revealed that the T-DNA is located at position + 507 within the AP2/ERF domain (Fig. S2a). RT-PCR analysis did not detect any expression of *ERF102* in *erf102* plants, suggesting that it is a null allele (Fig. S2b). The morphological phenotype of the *erf102* mutant described below (Fig. S3e) was fully complemented by introgression of the *35S:ERF102* gene (Fig. S2c‒S2f). In several available T-DNA insertion lines for *ERF103* (SALK_087356, GABI_085B06) or *ERF104* (SALK_024275, SALK_057720, SALK_152806) we detected residual *ERF* expression. Therefore, lines with a reduced *ERF103* or *ERF104* expression were constructed using artificial microRNAs (amiRNAs) (Schwab et al. 2006). Two independent, homozygous amiRNA expressing lines with the lowest residual expression of the target genes were selected for further experiments (Fig. S3a and Bolt et al. 2017). Moreover, lines overexpressing *ERF102* to *ERF104* under control of the CaMV *35S* promoter were constructed and two strongly expressing lines selected (Fig. S3b‒d).

**Fig. 5:**
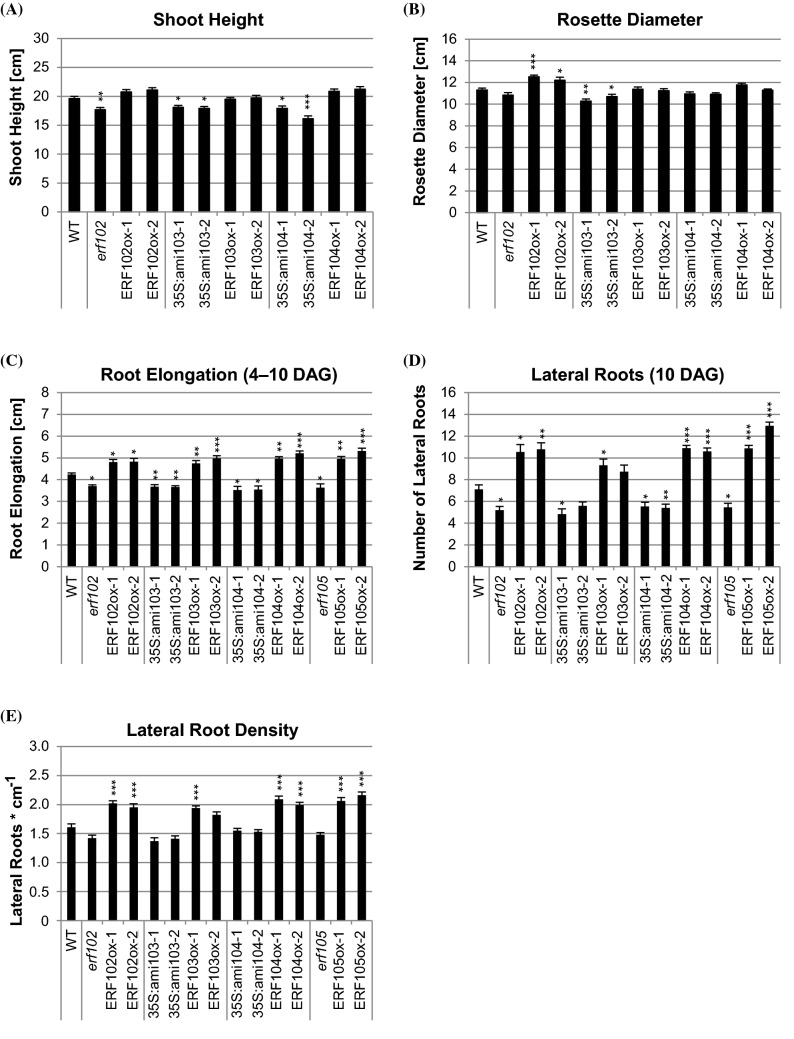
Shoot and root growth of lines with altered *ERF102* to *ERF105* expression levels. Shoot height (**a**) and rosette diameter (**b**) of 35-day-old plants grown on soil. (**c**) Elongation of the primary root determined between four and ten DAG (**c**), number of lateral roots (**d**) and lateral root density (**e**) determined ten DAG of plants grown on half-strength MS medium. Asterisks indicate significant differences to the wild type (n ≥ 30), (*p < 0.05; **p < 0.01; ***p < 0.001). Error bars represent SE

Morphological analysis of plants with reduced or increased *ERF102* to *ERF104* expression revealed in most cases only slight differences of shoot growth compared to wild-type plants. Furthermore, plants with altered expression of *ERF102*, *ERF103* or *ERF104* flowered at the same time as wild-type plants and showed a similar onset of leaf senescence (data not shown). In contrast, root elongation, the formation of lateral roots as well as the lateral root density was more strongly affected by altered expression of these genes (Fig. 5c‒e).

The *erf102* mutant exhibited an about 10% reduced shoot height compared to the wild type. Overexpressing lines of *ERF102* exhibited a slightly but not significantly increased shoot height as well as a 10% (ERF102ox-1) and 8% (ERF102ox-2) bigger rosette diameter (Fig. 5a, b). Moreover, ten DAG *erf102* exhibited 13% lower and ERF102ox lines 13% higher primary root elongation (Fig. 5c). *erf102* showed 27% less and ERF102ox-1 and ERF102ox-2 48% and 51% more lateral roots compared to wild type (Fig. 5d). Lateral root density was increased 29‒31% in the ERF102ox lines (Fig. 5e).

Both 35S:ami103 lines were smaller in size, with an 8% reduced shoot height and a 6‒9% reduced rosette diameter compared to the wild type, while *ERF103* overexpression did not cause phenotypic differences in shoot height and rosette size (Fig. 5a, b). Primary root elongation was about 13% lower in both 35S:ami103 lines whereas ERF103ox-1 and ERF103ox-2 exhibited 12% and 17% longer primary roots compared to wild type (Fig. 5c). Similarly, 35S*:*ami103 lines had up to 32% less and ERF103ox plants up to 31% more lateral roots than wild type (Fig. 5d).

35S*:*ami104 lines had a 9% (35S*:*ami104-1) and 18% (35S*:*ami104-2) reduced shoot height, but an unchanged rosette diameter (Fig. 5a, b). Primary root elongation of 35S:ami104 lines was about 13% slightly reduced and enhanced by up to 29% in *ERF104* overexpressing lines (Fig. 5c). The number of lateral roots was reduced by about 20% in both 35S*:*ami104 lines, while ERF104ox-1 and ERF104ox-2 exhibited 57% and 53% more lateral roots (Fig. 5d) and had a 30% and 22% higher lateral root density compared to wild type (Fig. 5e).

Bolt et al. (2017) described that the shoot phenotype of *erf105* and ERF105ox lines resembled the wild type. Here, root analysis revealed a 14% lower primary root elongation (Fig. 5c) and 23% less lateral roots in *erf105* compared to wild type (Fig. 5c). ERF105ox lines showed a 17‒25% higher primary root elongation, 53‒83% more lateral roots and a 31‒44% higher lateral root density compared to wild type (Fig. 5c‒e).

To examine a potentially redundant role of the four *ERF* genes, several higher order mutants were generated, namely *erf102 *35S:amiERF103, *erf102 *35S:amiERF104, *erf105 *35S*:*amiERF103, and *erf102* 35S*:*amiERF104/105. These lines include all possible combinations of at least two *ERF* genes that are mutated or have a lowered expression, except combined loss of function of *ERF103* and *ERF104*. Higher order mutants did not show a phenotypic additive effect compared to the respective single mutants with respect to rosette diameter, shoot height, primary root elongation, number of lateral roots and flowering time (data not shown). These results suggest that *ERF102* to *ERF105* are not acting redundantly on growth regulation. However, we cannot exclude that the degree of downregulation achieved by amiRNAs is insufficient to uncover redundant gene activities.

### Analysis of the functions of the *ERF102* to *ERF105* genes in the cold acclimation response

ERF105 is a positive regulator of *Arabidopsis* freezing tolerance and cold acclimation (Bolt et al. 2017). Therefore, we analysed whether the *ERF102* to *ERF104* genes, which are also regulated by cold (Lee et al. 2005; Vogel et al. 2005; Park et al. 2015; Bolt et al. 2017), also play a role in regulating freezing tolerance and cold acclimation. To this end, we studied the transcript accumulation of selected cold responsive genes in *ERF* single and double mutants and analysed the freezing tolerance of these mutants.

First, we examined the expression levels of selected cold-responsive genes in plants with reduced or enhanced expression of a single *ERF102* to *ERF104* gene before (non-acclimated, NA) and after 14 days of cold acclimation (ACC14) and compared these to wild type. The transcript levels of cold-responsive genes were in all lines similar to wild type (Fig. S4), which contrasts with the strongly altered transcript levels displayed by the *erf105* mutant and *ERF105* overexpressing lines (Bolt et al. 2017).

**Fig. 6:**
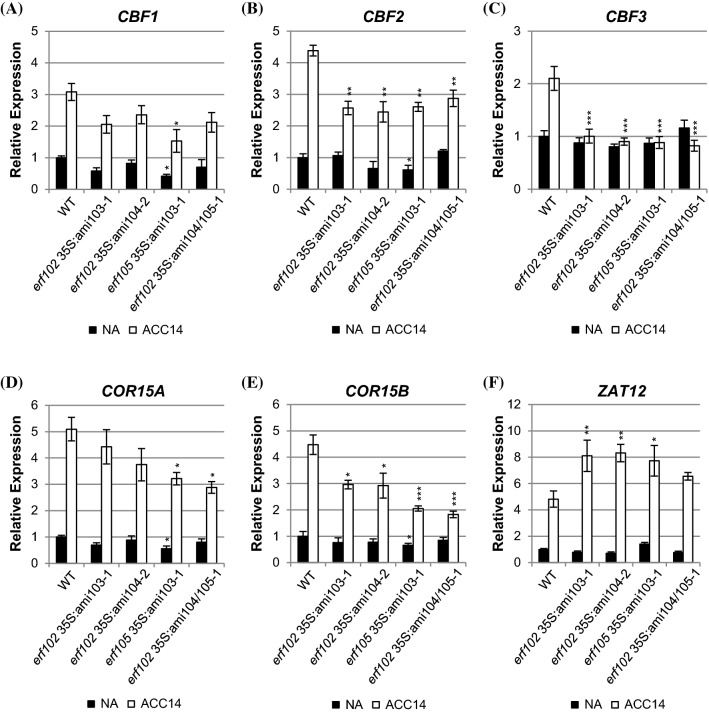
Expression of selected cold-responsive genes in lines with reduced *ERF102* to *ERF105* expression. Relative expression of *CBF1* (**a**), *CBF2* (**b**), *CBF3* (**c**), *COR15A* (**d**), *COR15B* (**e**) and *ZAT12* (**f**) genes in six-week-old lines with reduced *ERF102* to *ERF105* expression before (non-acclimated, NA) and after 14 days (acclimated, ACC14) of cold acclimation at 4 °C. Transcript levels of wild-type samples under non-acclimated conditions were set to 1 (n ≥ 4). Asterisks indicate significant differences to the respective wild-type condition (*p < 0.05; **p < 0.01; ***p < 0.001). Error bars represent SE

The analysis of higher order mutants revealed that under non-acclimated (NA) conditions the steady state mRNA levels of *CBF1*, *CBF2*, *COR15A*, and *COR15B* were up to 60% lower in the *erf105* 35S:ami103-1 plants compared to those of the wild type (Fig. 6). In all other mutant combinations the basic expression level of these cold-responsive genes was slightly, but not significantly lower than in the wild type. After 14 days of acclimation at 4 °C (ACC14), the expression levels of these genes were elevated between two- and five-fold in wild type compared to NA plants. ACC14 plants with mutated *ERF102* or *ERF105* genes combined with reduced expression of *ERF103* or *ERF104* showed, in most cases, a lower induction of the cold-responsive genes. For example, the induction levels of *CBF2* and *COR15B* were reduced in all hybrid lines to about 50% of the wild-type level. Strikingly, the induction of *CFB3* was completely absent in all mutant lines while it was induced about twofold in wild type. In contrast, *ZAT12* gene expression showed a stronger increase in *erf102* 35S*:*ami103-1, *erf102 *35S:ami104-2 and *erf105* 35S*:*ami103-1 than in wild type (Fig. 6f).

**Fig. 7:**
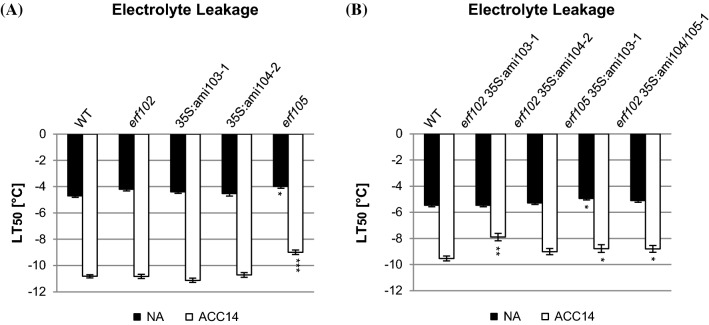
Electrolyte leakage assays of lines with reduced *ERF102* to *ERF105* expression. Electrolyte leakage assays on detached leaves of lines with mutations or reduced expression affecting single *ERF* genes (**a**) or several *ERF* genes (**b**) before (non-acclimated, NA) and after 14 days (acclimated, ACC14) of cold acclimation at 4 °C. The bars represent the means ± SE from four replicate measurements where each replicate comprised leaves from three plants. Asterisks indicate significant differences to the wild type (*p < 0.05; **p < 0.01; ***p < 0.001)

Next, we determined the freezing tolerance of plants with reduced *ERF102, ERF103* and *ERF104* gene expression before and after 14 day of cold acclimation at 4 °C by an electrolyte leakage assay of detached leaves (Rohde et al. 2004; Thalhammer et al. 2014). To take into account the almost complete arrest of plant growth at 4 °C, the electrolyte leakage assay was performed at the same developmental state for both NA and ACC plants. *erf105* mutant plants used as positive control showed higher LT_50_ (temperature of 50% electrolyte leakage) values (-3.99 ± 0.13 °C in NA plants and − 8.99 ± 0.17 °C in ACC14 plants) compared to wild type (− 4.7 ± 0.11 °C in NA plants and − 10.82 ± 0.12 °C in ACC14 plants) (Fig. 7a), which is consistent with previous results (Bolt et al. 2017). In contrast, *erf102*, 35S:ami103-1 and 35S:ami104-2 plants did not show differences in LT_50_ values compared to wild type. Also, overexpression of single *ERF102*, *ERF103* or *ERF104* genes did not lead to altered freezing tolerance under NA conditions (Fig. S5). The behavior of the overexpressing lines in response to acclimation was not tested.

Analysis of the freezing tolerance of higher order mutants revealed that only the *erf105* 35S*:*ami103-1 plants showed higher LT_50_ values (− 4.93 ± 0.12 °C) compared to wild type (− 5.46 ± 0.12 °C) under NA conditions (Fig. 7b). Following cold acclimation, several combinations exhibited higher LT_50_ values compared to wild type (− 9.54 ± 0.18 °C). The strongest change was shown by *erf102* 35S*:*ami103-1 (− 7.89 ± 0.24 °C), while *erf105 *35S*:*ami103-1 (− 8.78 ± 0.25 °C) as well as *erf102 *35S*:*ami104/105–1 (− 8.79 ± 0.25 °C) showed smaller effects. In contrast, *erf102* 35S:ami104-2 showed a similar LT_50_ as wild type after cold acclimation (Fig. 7b).

## Discussion

Recently, we reported that *ERF102* to *ERF105* are regulated by cold and different cold stress-related hormones, and we demonstrated that *ERF105* has a function in the freezing tolerance and cold acclimation of *Arabidopsis* (Bolt et al. 2017). In the present study we significantly extended this work and first investigated further expression characteristics of the gene family members and then explored their potentially redundant roles in regulating plant growth and the cold acclimation response.

### The *ERF102* to *ERF105* genes show overlapping expression patterns

The similar profiles of gene expression in response to hormone or stress treatment are consistent with a partial functional redundancy of *ERF102* to *ERF105*. For instance, all genes were rapidly downregulated by SA (Fig. 2b) and upregulated by high light or H_2_O_2_ (Fig. 2e, f). Network analysis of publicly available transcriptome data using for instance GeneMANIA (Warde-Farley et al. 2010) also showed that these four *ERF* genes are co-regulated and co-expressed in a large number of conditions including numerous hormone and chemical treatments (Fig. S6). However, some individual response profiles were discovered as well. Thus, not all four *ERF* genes were transcriptionally regulated by heat, drought, NaCl, or mannitol (Fig. 2). Together, the analysis of transcriptional regulation and the occurrence of *cis-*acting promoter sequences is in line with the idea that *ERF102* to *ERF105* have roles in multiple hormone and stress responses as was shown for these and other ERFs in a number of cases (Bethke et al. 2009; Moffat et al. 2012; Son et al. 2012; Dubois et al. 2013; reviewed by Licausi et al. 2013; Mase et al. 2013; Meng et al. 2013; Sewelam et al. 2013; Moore et al. 2014a, b; Vogel et al. 2014; Dubois et al. 2015; Xie et al. 2019).

### The *ERF102* to *ERF105* genes have a limited impact on plant growth

The tissue-specific expression patterns of *pERF102:GUS* to *pERF105:GUS* are partly overlapping, which is in accordance with a redundant function of the ERF proteins. All four genes are predominantly expressed in the root, only for *pERF105:GUS* a significant expression was detected also in several shoot tissues such as vasculature, apical shoot and stomata (Bolt et al. 2017). Expression of all four *pERF-GUS* reporter genes was visible shortly after germination in different cell types of the radicle and later in distinct root tissues and cell types. For example, *pERF102:GUS*, *pERF103:GUS* and *pERF105:GUS* were expressed in the cortex cells that surround emerging lateral roots. Interestingly, expression of *ERF102*, *ERF103* and *ERF105* is regulated by cytokinin and auxin, two key hormones of lateral root development (Benková et al. 2003; Casimiro et al. 2003; Swarup et al. 2008; Chang et al. 2013, 2015). Insertional mutants and amiRNA lines indicated a role of these genes in regulating root elongation. All mutants (*erf102*, *erf105*) as well as 35S:ami103 and 35S:ami104 lines had about 10–15% shorter roots and all overexpressing showed a ca. 20% increased root elongation (Fig. 5c). Most loss-of-function mutants formed also less lateral roots. However, the differences were small and the lateral root density mostly not significantly altered (Fig. 5d‒e). Opposite and stronger phenotypic changes were noted in the respective overexpressing lines, which had an increased number (by up to ~ 85%) of lateral roots and a higher lateral root density. Although overexpression experiments may produce artefacts and are not fully conclusive they have been often informative about the functional context of a given gene. Loss-of-function phenotypes of genes regulating root architecture can be subtle or depend on the environmental or developmental context (Motte et al. 2019) and thus might have gone unnoticed in the *erf* mutants. The strong regulation of the four *ERF* genes by different stressors suggests that they might be particularly relevant under stressful conditions. It cannot be excluded that members of the *ERF105* gene subfamily studied here contribute to regulating root architecture under specific environmental conditions, this requires further investigation.

Among the expression sites of the four *ERF* genes, the expression of *pERF104:GUS* in the quiescent center (Fig. 3r) particularly intriguing. Noteworthy, among the direct targets of ERF104 is the transcription factor gene *SCARECROW* (*SCR*) (Sparks et al. 2016). SCR is, together with SHORTROOT, essential for quiescent center specification and maintenance (reviewed by Benfey 2016; Salvi et al. 2018). Further, in a yeast two-hybrid screen the transcription factor MYB56/BRASSINOSTEROIDS AT VASCULAR AND ORGANIZING CENTER (BRAVO) was identified as an interactor of ERF104 (our unpublished result). MYB56/BRAVO represses cell divisions in the quiescent center thus counteracting SCR (Di Laurenzio et al. 1996; Vilarrasa-Blasi et al. 2014). It is known that interaction with other transcription factors modulates the activity of ERFs (Licausi et al. 2013; Xie et al. 2019). While these data suggest that ERF104 might be part of the transcription factor network in the quiescent center, we have been unable to detect any changes of cellular organisation in the quiescent center and surrounding cells nor did we detect altered *SCR* gene expression in the 35S:ami104 and ERF104ox lines (data not shown). It could be that the decrease in *ERF104* expression obtained in the amiRNA lines is not sufficient to cause a strong loss-of-function phenotype, analysis of a null mutation could be more informative.

### The *ERF102* to *ERF105* genes regulate the response to cold stress

One important goal of this work was to analyse the possible roles of the ERF105-related transcription factors in the response to cold stress. *ERF102* to *ERF105* are rapidly cold-induced (Bolt et al. 2017) in parallel with the first wave transcription factors of the cold stress response including the *CBF* genes (Park et al. 2015). Mutation or reduced expression of *ERF102, ERF103* or *ERF104* single genes did not lead to an altered freezing tolerance. In case of the amiRNA lines this could be due to residual gene expression (Figs. 7a and S1). Thus, among the four genes only the mutation of *ERF105* resulted in a decreased freezing tolerance before and after cold acclimation compared to wild type underpinning its primary role (Fig. 7a and Bolt et al. 2017). However, the analysis of freezing tolerance of higher order mutants indicated that *ERF102* and *ERF103* also play a role in cold acclimation, since the reduced expression of both genes resulted in altered expression of cold response genes (Fig. 6) and higher freezing sensitivity (Fig. 7b). The eventual role of ERF104 cannot be determined with certainty as only amiRNA lines were available and not all combinations with other *ERF* genes were tested. 35S:ami104 lines in combination with the *erf102* mutation showed an altered expression of cold-responsive genes similar to other double mutant combinations (Fig. 6) and the LT_50_ value was higher than in wild type although the significance was below the threshold (p < 0.05), indicating that ERF104 might be involved in the response to cold as well. Our attempts to demonstrate a role of these *ERF* genes at low temperatures in the root as was reported for *CRF2* and *CRF3* belonging to a different class of *ERF* genes (Jeon et al. 2016), have failed. Such an activity could, as was stated above, be masked by incomplete loss of function and/or the unknown nature of their specific activities.

Based on transcript data which show a lowered activation of *CBF* and *COR* genes in *erf* gene mutants after cold acclimation (Fig. 6), ERF102, ERF103 and ERF104 may also play a role upstream of these genes as was suggested for ERF105 (Bolt et al. 2017). Increased *CBF3* expression upon cold acclimation was even completely lacking in the *erf* mutants (Fig. 6c) but the gene was still cold responsive at earlier time points although with a reduced amplitude as compared to wild type (Fig. S7). A proximity of the four *ERF* genes to the CBF regulon was also suggested by the result of the network analysis which placed several proteins that are part of the CBF regulon (CBF2/DREB1c, ZAT10 und RAP2.13/RAP2.4) in the vicinity of ERF102 to ERF105 (Fig. S6).

The lower activation of the *CBF* and *COR* genes in cold-acclimated *erf* gene mutants could be at least partially due to enhanced expression of another gene belonging to the CBF regulon, *ZAT12* (Fig. 6f). *ZAT12* encodes a zinc-finger protein known to be a negative regulator of the CBF regulon and is usually induced in parallel with *CBF* and *COR* genes providing a negative regulatory feedback loop (Vogel et al. 2005). The higher expression of *ZAT12* in the *erf* higher order mutants suggests that these *ERF* genes may act as negative regulators of *ZAT12* expression and in this way as positive regulators of *CBF* and *COR* genes. Notably, the *ZAT12* gene does not possess the specific DNA-binding motif of ERF transcription factors, the GCC-box, in its promoter region (Hao et al. 1998) suggesting that additional factors might be required for its repression by ERFs.

Knockout/knockdown of single *ERF102* to *ERF104* genes did not cause an altered transcript level of cold-responsive genes after 14 day of cold acclimation (Fig. S4), which is again in line with the assumption that these *ERF* genes may have redundant roles. Lines overexpressing *ERF102* to *ERF104* did neither show a differential expression of cold-responsive genes nor an altered freezing tolerance (Fig. S4 and S5), similar to *ERF105* overexpressing lines (Bolt et al. 2017). It is possible that ERF102 to ERF105 are required for the transcriptional activation of these target genes but are not the rate-limiting factors, for example because they function as part of a complex. Alternatively, activity of these proteins under cold may depend on additional regulatory steps such as phosphorylation which could be transient. Indeed, the phosphorylation of ERF102 to ERF104 by MPK3 and/or MPK6 was shown (Bethke et al. 2009; Son et al. 2012; Wang et al. 2013) and functions of MPK3 and MPK6 in the cold signalling pathway have been described (Kim et al. 2017; Li et al. 2017; Zhao et al. 2017).

Taken together, the data document similar and possibly partially redundant functions of *ERF102* to *ERF105* in response to cold. Notably, combined action of related *ERF* transcription factor genes has also been reported in other cases (Jeon et al. 2016; Kim et al. 2016). Future work will investigate how the ERF102 to ERF105 proteins are integrated in the extensive transcriptional network governing the response to cold (Park et al. 2015; Jia et al. 2016; Zhao et al. 2016).

## Methods

### Plant material

*Arabidopsis thaliana* accession Col-0 was used as wild type. The *erf105* mutant, *ERF105* overexpressing lines, *pERF105:GUS* lines, complementation lines of *erf105,* as well as 35S:ami104 and 35S:ami104/105 lines have been described previously (Bolt et al. 2017). The T-DNA insertion line *erf102* (SAIL_46_C02) was obtained from the Nottingham Arabidopsis Stock Centre (NASC). After selection of homozygous plants, the location of the T-DNA insertion was verified by sequencing and plants were backcrossed twice with Col-0 to eliminate possible multiple insertions and other background mutations. Complementation of the *erf102* phenotype was tested by introgressing ERF102ox-1 and ERF102ox-2 into the *erf102* background. To generate lines overexpressing *ERF102* to *ERF104*, the genomic coding sequences of *ERF102* to *ERF104* were amplified by PCR, cloned into pDONR221 (Invitrogen, Carlsbad, USA) by using the Gateway cloning system and transferred subsequently into vector pK7WGF2 (Karimi et al. 2007b). To generate *pERF102:GUS* to *pERF104:GUS* reporter genes, the promoter regions of the *ERF* genes (~ 2 kb upstream of the start codon) were amplified by PCR and cloned into pDONR P4-P1R (Invitrogen). To generate the binary destination vectors, the pDONR P4-P1R constructs with the *ERF* promoters and the Gateway entry clone pEN-L1-SI-L2 (Karimi et al. 2007a) harboring the *GUS* reporter gene were then combined into the destination vector pK7m24GW,3 using MultiSite Gateway (Karimi et al. 2005). Artificial microRNA (amiRNA) was used to generate lines with a reduced *ERF103* expression (Schwab et al. 2006). amiRNAs directed against *ERF104* and *ERF105* were described (Bolt et al. 2017). The amiRNA sequence targeting *ERF103* was 5′-TAACGTCGTAACTTTCCCCCG-3′. The sequence was selected and the expression construct was made using the Web MicroRNA Designer (WMD3) and the protocol available under https://wmd3.weigelworld.org. The amiRNA precursor was cloned into pDONR221 (Invitrogen) and subsequently into pH2GW7 (Karimi et al. 2007b) harboring the cauliflower mosaic virus (CaMV) *35S* promoter to yield 35S:ami103. All primers used for cloning are listed in Table S1. The binary constructs were transformed into Col-0 plants by *Agrobacterium tumefaciens* (GV3101:pMP90) using the floral dip method as described by Davis et al. (2009). Higher order mutants with reduced expression of *ERF* genes were generated by crossing amiRNA lines with T-DNA insertion lines.

### Growth conditions, hormone and stress treatment

For hormone and stress treatments, plants were grown in vitro under long day (LD) conditions (16 h light/8 h dark) and 21 °C in half strength liquid Murashige and Skoog (MS) medium (for hormone treatment) or on solid MS medium (for stress treatment), in each case containing 0.1% sucrose (Murashige and Skoog 1962). Eleven days after germination (DAG), hormonal treatments were performed by adding the respective hormone to the liquid medium. Seedlings grown on solid medium were exposed to different stress treatments eleven DAG, including heat treatment at 42 °C in darkness, high light stress (1000 µmol m^−2^ s^−1^) instead of standard light (100‒150 µmol m^−2^ s^−1^), oxidative stress by spraying seedlings with 500 mM H_2_O_2_ or transferring seedlings to liquid medium with 30 µM paraquat, drought stress by transferring seedlings to dry filter paper, or salt/osmotic stress by transplanting seedlings to MS medium including 200 mM NaCl or 200 mM mannitol, respectively, for different time periods. Control plants were treated with the respective control conditions, which were the respective mock solution in the hormone experiment, 21 °C in the heat stress experiment, standard light conditions in the high light experiment, spraying with mock solution in the H_2_O_2_ experiment, and transferring to moist filter paper in the drought experiment, or mock medium in the paraquat, salt and osmotic stress experiments.

For the analysis of growth and developmental parameters, plants were grown on soil in the greenhouse under LD conditions (16 h light/8 h dark) at a light intensity of 130‒160 µmol m^−2^ s^−1^ and 21 °C. Fourteen, 21, 28, and 35 DAG rosette diameter and shoot height were determined. Furthermore, the flowering time, defined as opening of the first flower, was recorded. Leaf senescence was recorded based on visual inspection of the oldest leaves turning yellow.

For analysis of roots, plants were grown in vitro in vertically placed square petri dishes on half strength MS medium containing 10 g L^−1^ phytagel. The elongation of the primary root was determined from digital images between four and ten DAG using the software ImageJ (Abràmoff et al. 2004). The number of lateral roots was determined ten DAG from the same images.

For electrolyte leakage experiments, plants were grown for two weeks under SD conditions and then for four weeks under LD conditions at 200 µmol m^−2^ s^−1^ and 20 °C during the day, 18 °C during the night (non-acclimated plants). For cold acclimation, plants were transferred to a cold chamber and cultivated under LD (90 µmol m^−2^ s^−1^) at 4 °C for additional 14 days.

### RNA analysis

Total RNA was extracted from tissues using the NucleoSpin RNA Plant Kit (Macherey & Nagel, Düren, Germany) according to the manufacturer’s instructions, including an on-column DNase digestion. As a control, quantitative real-time PCR (qRT-PCR) measurements using intron-specific primers for AT5G65080 were performed to confirm the absence of genomic DNA contamination (Zuther et al. 2012). For RT-PCR, 500 ng RNA were reverse transcribed using the QIAGEN OneStep RT-PCR Kit according to the manufacturer’s information (Qiagen, Hilden, Germany). The sequences of primers were as follows: *Actin2-*F, 5′-TACAACGAGCTTCGTGTTGC-3′; *Actin2-*R, 5′-GATTGATCCTCCGATCCAGA-3′; *ERF102*-F, 5′-CTGCACTTTGGTTCATCGAG-3′; *ERF102*-R, 5′-GAGATAACGGCGACAGAAGC-3′. For qRT-PCR analyses, 1 µg RNA was transcribed into cDNA by SuperScript III Reverse Transcriptase (Invitrogen) according to the manufacturer’s instructions using a combination of oligo(dT) primers and random hexamers. qRT-PCR analyses were performed as previously described by Bolt et al. (2017). Four biological replicates were used and each qRT-PCR experiment was performed twice. In all cases both experiments yielded similar results and one result is shown exemplarily.

### GUS staining and microscopy

Histochemical analysis to detect GUS reporter enzyme activity was performed as described by Jefferson et al. (1987) with some modifications as described by Bolt et al. (2017). GUS analyses were carried out with two or three independent *pERF:GUS* lines for each of the constructs and identical expression patterns were seen. The histochemical analyses were repeated several times with plants of different age.

### Promoter sequence analysis

Analysis of the *ERF102* to *ERF105* promoters for the presence of *cis*-acting regulatory elements (CAREs) was carried out using the PLACE website (https://www.dna.affrc.go.jp/PLACE/; Higo et al. 1998). The MotifMapper of the TOUCAN2 workbench (Aerts et al. 2005) was used to determine the frequency of CAREs in 2 kb of the *ERF102* to *ERF105* promoters (*pERF102* to *pERF105*).

### Transient gene expression in *Nicotiana benthamiana* and confocal laser scanning microscopy

Subcellular localisation of GFP fused to ERF proteins was done in leaves of 6-week-old *N. benthamiana* according to Sparkes et al. (2006) with the equipment described by Bolt et al. (2017).

### Electrolyte leakage

Electrolyte leakage was determined with detached leaves over a temperature range from − 1 to − 16 °C for non-acclimated plants and from − 2 to − 22 °C for cold acclimated plants, cooled at a rate of 4 °C h^−1^ as described by detail in Rohde et al. (2004) and Thalhammer et al. (2014). Four technical replicates were analysed for each temperature point, and for each of these replicates leaves from three different plants were pooled. The temperature of 50% electrolyte leakage (LT_50_) was calculated as the log EC50 value of sigmoidal curves fitted to the leakage values using the software GraphPad Prism3 (GraphPad Software, Inc., La Jolla, USA).

### Statistical analyses

Every experiment was conducted at least twice. Figures show data of a single experiment that is representative of two or three experiments showing similar results. Data are presented as the mean ± standard error. Statistical analyses were performed using SAS or GraphPad Instat Software (one-way ANOVA or two-way repeated measures ANOVA with Tukey’s post hoc test). Normality and homogeneity of variance were tested using the Shapiro–Wilk and Levene tests (Neter et al. 1996). In order to meet the assumptions, data sets were transformed using log or square-root transformation. If assumptions were not met, a nonparametric Kruskal–Wallis test was carried out followed by a Mann–Whitney test to perform a pairwise comparison.

## Electronic supplementary material

Below is the link to the electronic supplementary material.

Supplementary file1 (PDF 1207 kb)
