# Supplementary material for: Characterisation of the ERF102 to ERF105 genes of Arabidopsis thaliana and their role in the response to cold stress
Source: Plant Mol Biol. 2020 Mar 18;103(3):303–20. doi: 10.1007/s11103-020-00993-1 (PMC7220888; doi:10.1007/s11103-020-00993-1)
Supplement: Supplementary file 1 — Supplementary file1 (PDF 1207 kb) [file 11103_2020_993_MOESM1_ESM.pdf]

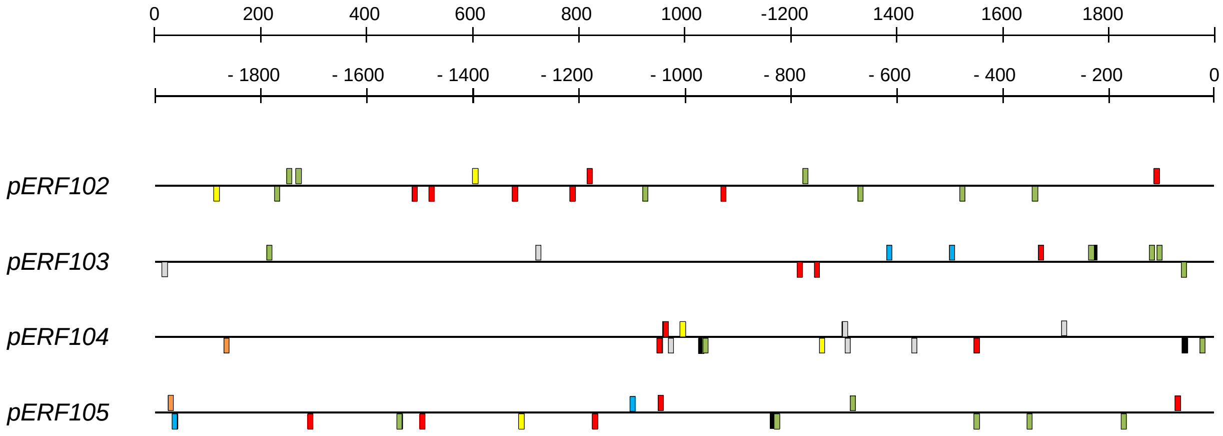

- ASF1MOTIFCAMV (TGACG, ASF-1 binding site, related to auxin, salicylic acid and light regulation; Despres et al., 2003)
- WBOXATNPR1 (TTGAC, salicylic acid and biotic stress-related element; Chen & Chen, 2002)
- CCAATBOX1 (CCAAT, heat stress-related element; Haralampidis et al., 2002)
- ROSE3 (ATCCAAA, ROS-responsive element 3; Wang et al., 2013)
- ROSE5 (TAATTTGG, ROS-responsive element 5; Wang et al., 2013)
- MYB2AT (TAACTG, binding site for MYB2, water stress-related; Urao et al., 1993)
- MYCATRD22 (CACATG, drought- and abscisic acid response-related element; Chinnusamy et al., 2003)

Fig. S1 *cis*-acting regulatory elements in the *ERF102* to *ERF105* promoters. Analysis of the *ERF102* to *ERF105* promoters for the presence of *cis*-acting regulatory elements (CAREs) was carried out using the PLACE website (http://www.dna.affrc.go.jp/PLACE/; Higo et al., 1998). The motifmapper of the TOUCAN2 workbench (Aerts et al., 2005) was used to visualize the location of CAREs. The 2 kb promoter regions are represented by black lines. Colored boxes shown above the black lines represent CAREs oriented in the 5' → 3’ direction, colored boxes below the black lines indicate CAREs in the 3' → 5’ direction. A selection of CAREs relevant for gene regulation by hormonal or environmental cues shown in Fig. 2 is shown. Additional CAREs found in the *ERF102* to *ERF105* promoters are shown in Table S2

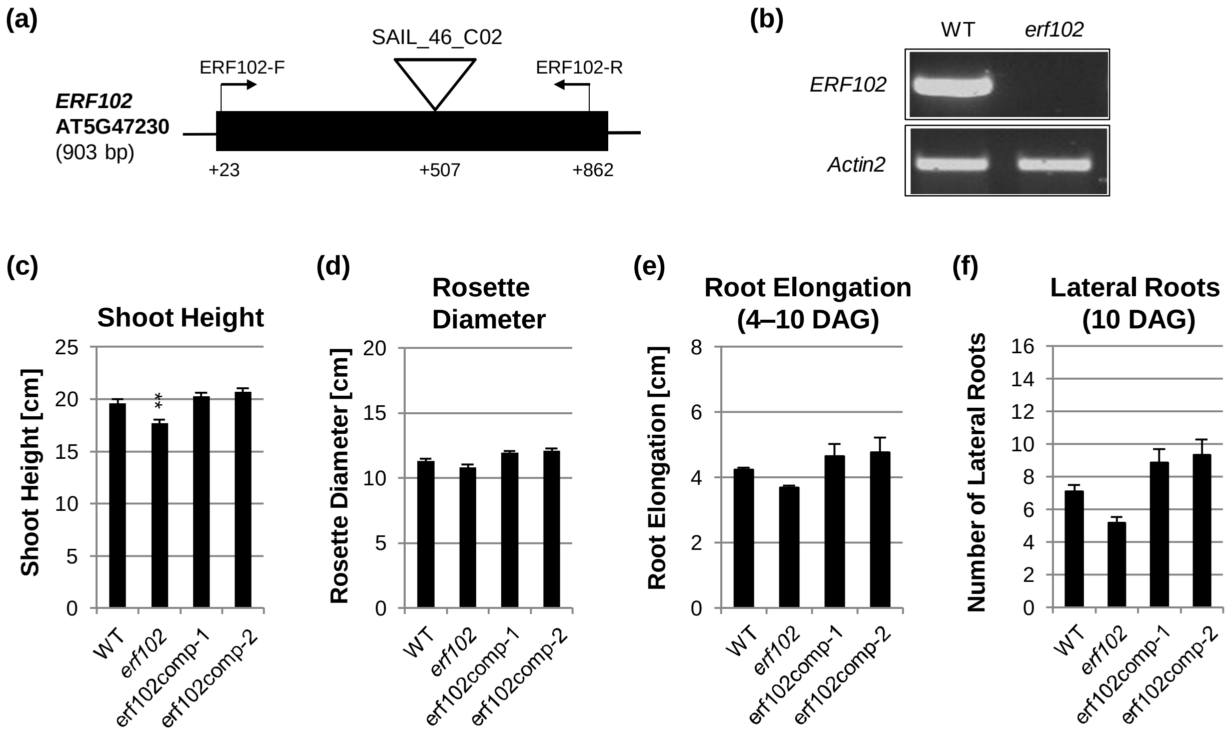

Fig. S2 Characterisation of the *erf102* mutant SAIL_46_C02. (**a**) Structure of the *Arabidopsis ERF102* (AT5G47230) gene. The black line denotes the untranslated region, the black box represents the exon, the T-DNA insertion at position +507 is shown by a triangle. The positions of primers that were used for RT-PCR are indicated by arrows. (**b**) RT-PCR analysis of *ERF102* expression using total RNA extracted from seedlings of wild type and *erf102*. The *Actin2* gene was used as internal control. (**c–f**) Complementation of the *erf102* mutant by introgression of the *35S:ERF102* gene. Shoot height (**c**) and rosette diameter (**d**) of 35-day-old plants. (**e**) Elongation of the primary root and (**f**) number of lateral roots of plants grown on half-strength MS medium. Asterisks indicate significant differences to the wild type ($n \geq 30$), (*, $p < 0.05$; **, $p < 0.01$). Error bars represent SE

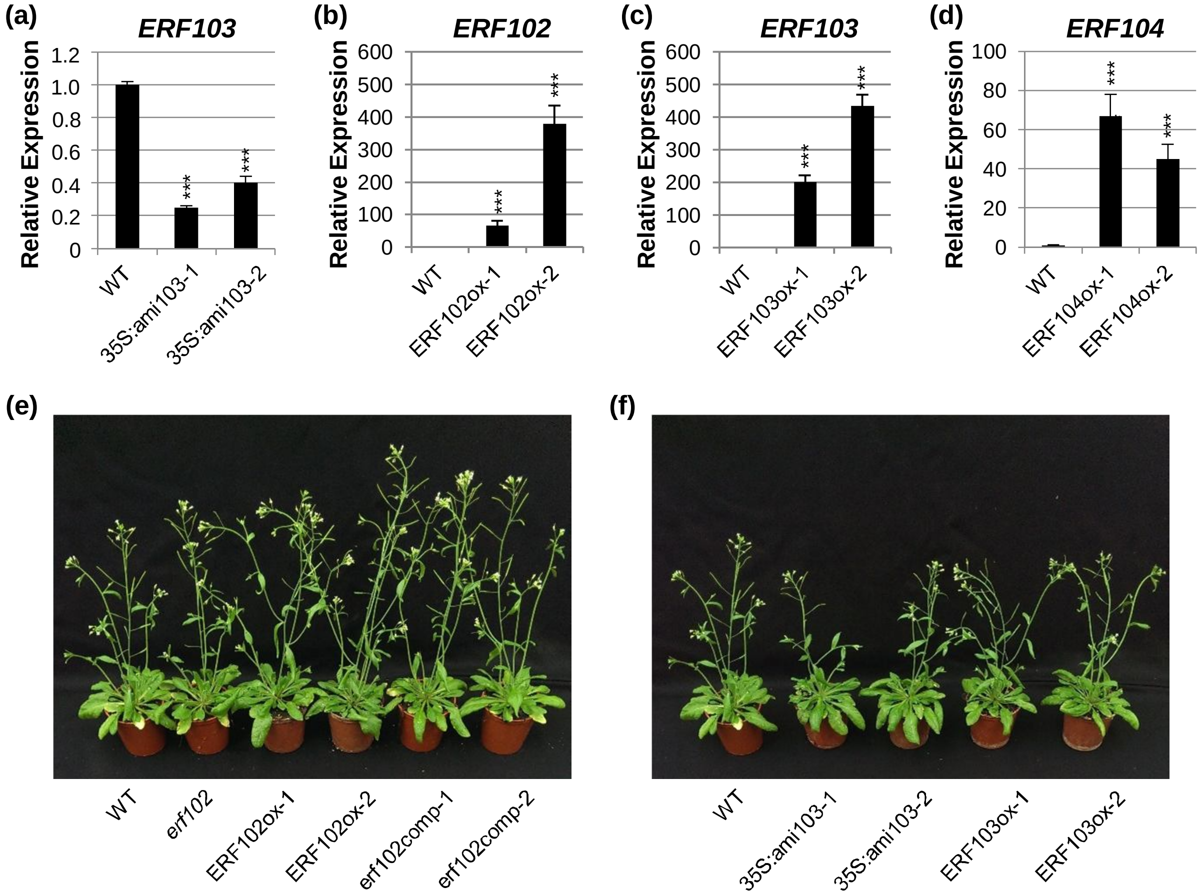

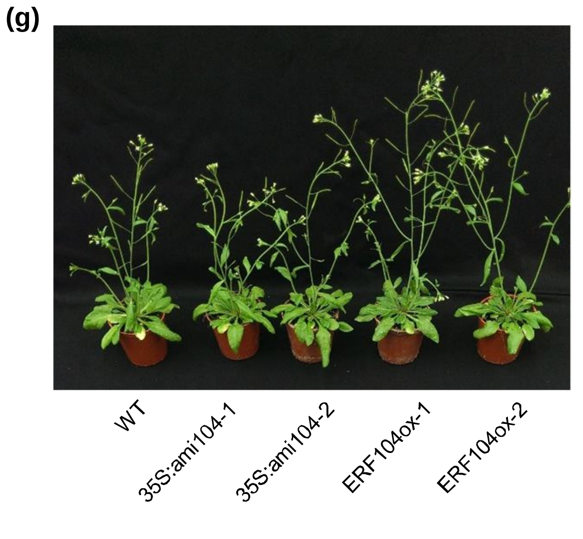

Fig. S3 Analysis of lines with altered *ERF102* to *ERF104* expression levels. (**a–d**) Relative expression level of *ERF* genes in eight pooled eleven-day-old seedlings of wild type, lines expressing amiRNA directed against *ERF103* (**a**) and lines overexpressing *ERF102* (**b**), *ERF103* (**c**) or *ERF104* (**d**). Transcript levels of wild-type samples were set to 1 ($n \geq 4$). Asterisks indicate significant differences to the wild type (***, $p < 0.001$). Error bars represent SE. (**e–g**) Shoot phenotype of plants grown 35 days under long day conditions. The pictures are complementary to the data shown in Figure 5a and 5b

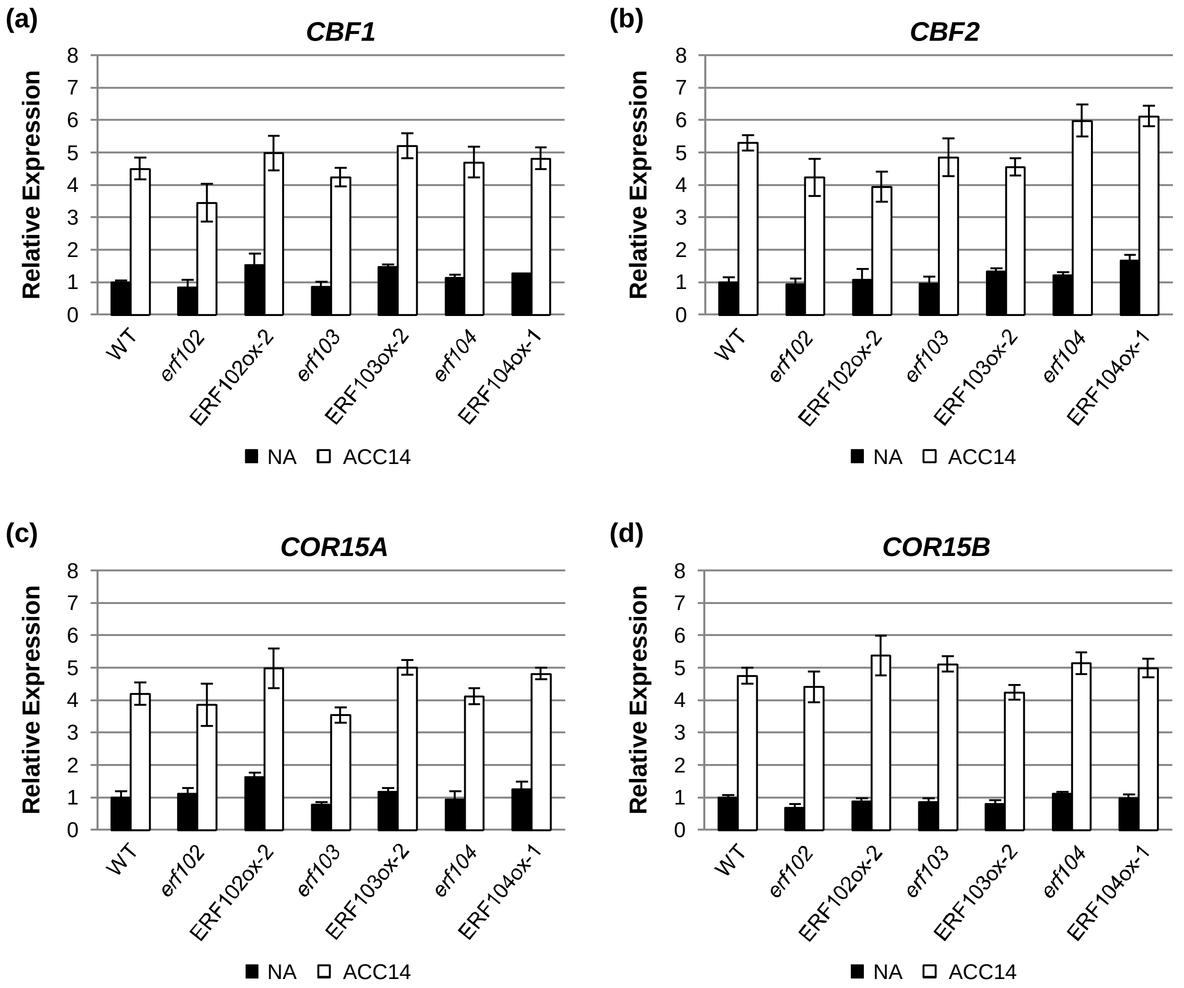

Fig. S4 Expression of selected cold-responsive genes in lines with reduced or enhanced *ERF102* to *ERF104* expression. Relative expression of *CBF1* (**a**), *CBF2* (**b**), *COR15A* (**c**), and *COR15B* (**d**) genes in six-week-old lines with reduced or enhanced *ERF102* to *ERF104* expression before (non-acclimated, NA) and after 14 days (acclimated, ACC14) of cold acclimation at 4 °C. Transcript levels of wild-type samples under non-acclimated conditions were set to 1 (n ≥ 4). Error bars represent SE

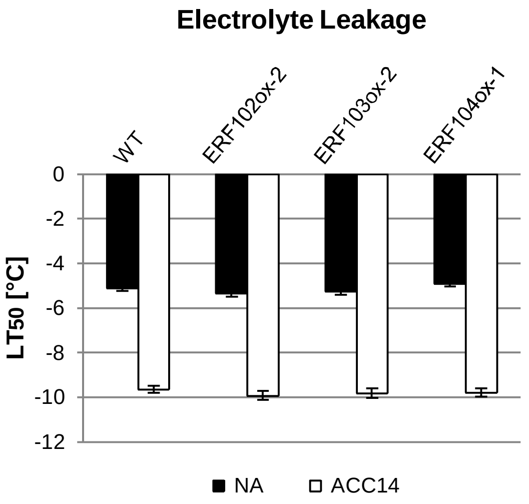

Fig. S5 Electrolyte leakage assays of lines with enhanced *ERF102* to *ERF104* expression. Electrolyte leakage assays on detached leaves of lines overexpressing *ERF102*, *ERF103* or *ERF104* before (non-acclimated, NA) and after 14 days (acclimated, ACC14) of cold acclimation at 4 °C. The bars represent the means ± SE from four replicate measurements where each replicate comprised leaves from three plants

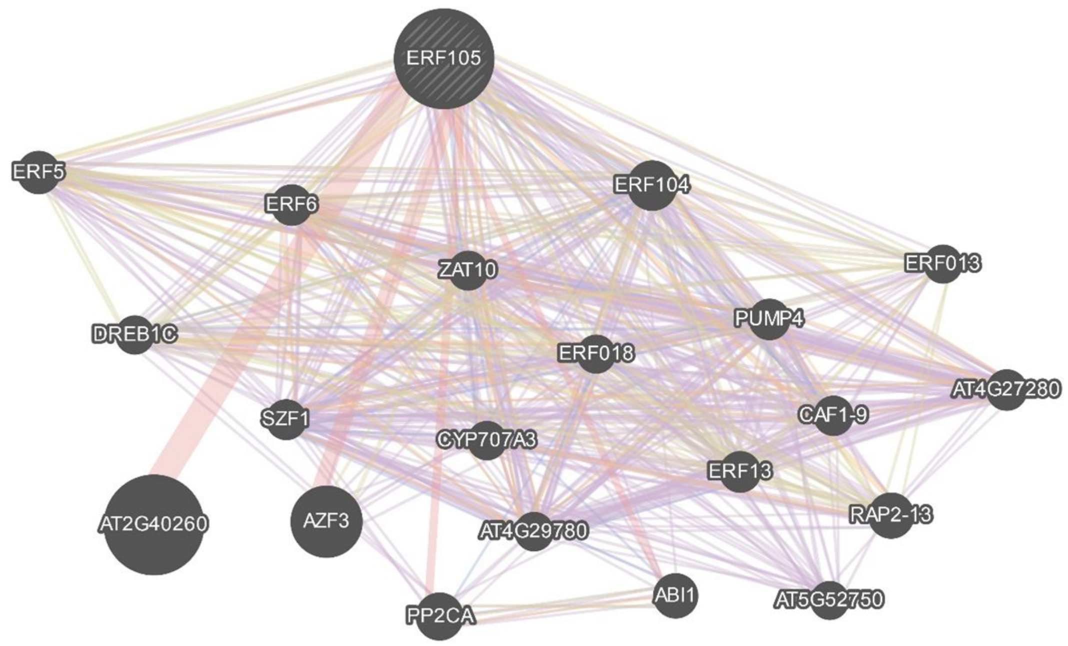

Fig. S6 Network of co-localisation, co-expression, genetic and physical interactions of ERF105. The blue connecting lines between two genes represent co-localisation, purple lines co-expression, green lines genetic interactions and red lines physical interactions. ABI1 = ABA INSENSITIVE 1; AZF3 = ZINC-FINGER PROTEIN 3; CAF1-9 = CCR4-ASSOCIATED FACTOR 1 HOMOLOG 9; CYP707A3 = CYTOCHROME P450, FAMILY 707, SUBFAMILY A, POLYPEPTIDE 3; DREB1C (CBF2) = DEHYDRATION-RESPONSE ELEMENT-BINDING PROTEIN 1C/C-REPEAT-BINDING FACTOR 2; ERF = ETHYLENE RESPONSE FACTOR, PP2CA = PROTEIN PHOSPHATASE 2CA; PUMP4 = PLANT UNCOUPLING MITOCHONDRIAL PROTEIN 4; RAP2-13 (RAP2.4/WIND) = RELATED TO AP2 13; SZF1 = SALT-INDUCIBLE ZINC-FINGER; ZAT10 (STZ) = ZINC FINGER PROTEIN 10 (SALT TOLERANCE ZINC FINGER). Analysis was done using GeneMania (Warde-Farley et al., 2010)

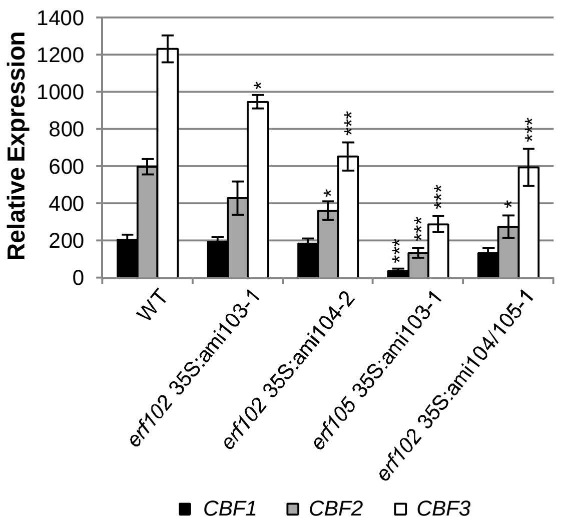

Fig. S7 Expression of selected cold-responsive genes in lines with reduced *ERF102* to *ERF105* expression. Relative expression of *CBF1*, *CBF2* and *CFB3* genes in eleven-day-old seedlings of wild type and lines with reduced *ERF102* to *ERF105* after 4 hours of cold treatment at 4 °C. Transcript levels of wild-type samples under control conditions were set to 1 ($n \geq 4$). Asterisks indicate significant differences to the wild type (*, $p < 0.05$; ***, $p < 0.001$). Error bars represent SE

Table S2 Sequences of primers used for cloning. Small letters in the primer sequences indicate the integrated *attB4*- or *attB1*-sites for cloning DNA fragments into the vector pDONR P4-P1R. Small italic letters in the primer sequences indicate the integrated *attB1*- or *attB2*-sites for cloning DNA fragments into the vector pDONR221. Underlined letters are the nucleotides added to keep the sequence in the right frame

Amplification of	Primer sequences (5'–3')
Promoter	F: ggggacaactttgtatagaaaagttgCGTTGATTCATTCTACAAACCAG
of *ERF102* (AT5G47230)	R: ggggactgctttttgtacaaacttgTGATAAAATTTTCAAAAAGC
Promoter	F: ggggacaactttgtatagaaaagttgGTTGTGGATTCTGGCATTTG
of *ERF103* (AT4G17490)	R: ggggactgctttttgtacaaacttgTTTGGAGGAAACAGAGAATTG
Promoter	F: ggggacaactttgtatagaaaagttgTGATGAGTGGTCGCTTCTTT
of *ERF104* (AT5G61600)	R: ggggactgctttttgtacaaacttgCTTCACTCTACTTGATTGAC
ERF102 (AT5G47230)	F: *ggggacaagtttgtacaaaaaagcaggct*CAATGGCGACTCCTAACGAAG
for N-terminal fusion	R: *ggggaccactttgtacaagaaagctgggt*GTAATCAAACAACGGTCAACTGG
ERF103 (AT4G17490)	F: *ggggacaagtttgtacaaaaaagcaggct*CGGAAATGGCTACACCAAACGAA
for N-terminal fusion	R: *ggggaccactttgtacaagaaagctgggt*CTCAAACAACGGTCAATTGTG
ERF104 (AT5G61600)	F: *ggggacaagtttgtacaaaaaagcaggct*CGATGGCAACTAAACAAGAAGCTTTA
for N-terminal fusion	R: *ggggaccactttgtacaagaaagctgggt*CGTTTTAAGTGACGGAGATAACGGAAA
